# Detection of honey adulteration using machine learning

**DOI:** 10.1371/journal.pdig.0000536

**Published:** 2024-06-10

**Authors:** Esmael Ahmed

**Affiliations:** Information System, College of Informatics, Wollo University, Dessie, Ethiopia; Amity University - Mumbai Campus, INDIA

## Abstract

Honey adulteration is a growing concern due to its health benefits and high nutritional content. Traditional methods like Melissopalynology are ineffective in detecting adulterated honey. This research presents a comparative study of machine learning algorithms for detecting adulteration in honey. The study uses hyperspectral imaging, a promising tool for food quality assurance, to classify and predict adulteration in honey. The proposed model relies on hyper-spectrum images and improves the accuracy of existing models using hyperparameter tuning. The dataset used includes segmented and pre-processed hyperspectral images of adulterated honey samples. The study found that machine learning and hyperspectral imaging can accurately identify if honey has been adulterated, with over 98% classification accuracy. The results showed that between 5% and 10% of adulterated honey samples are misclassified, with C1 Clover honey being the most frequently misclassified. This study aims to develop an efficient and accurate honey counterfeit detection technology using machine learning technologies such as Artificial Neural Networks (ANN), Support-vector machines (SVM), K Nearest Neighbors, Random Forests, and Decision trees. The proposed model relies on hyper-spectrum images and overcomes generalization to unknown honey types of problems. The dataset used includes segmented and pre-processed hyperspectral images of adulterated honey samples from seven different brands with 12 different botanical origin labels. Feature reduction techniques, such as feature ranking-based feature selection, and autoencoder techniques are employed to classify the botanical origins of honey. The model parameters are enhanced or tuned by the training process, and hyperparameters are adjusted by running the whole training data. The researchers used Python, and well-known algorithms like ANN, SVM, KNN, random forests, and decision trees. The results show that machine learning and hyperspectral imaging can accurately identify if honey has been adulterated, with over 98% classification accuracy.

## Author summary

Honey adulteration, the practice of adding substances such as sugar syrups to honey, poses significant challenges to food safety and consumer trust. In our study, we employ machine learning and hyperspectral imaging to develop a robust method for detecting adulterated honey. Using advanced algorithms, including artificial neural networks (ANN), support vector machines (SVM), K-nearest neighbors (KNN), random forests, and decision trees, we analyze honey samples to identify and quantify adulteration.

Our findings demonstrate that these machine learning techniques, particularly when enhanced through hyperparameter tuning, achieve high classification accuracy, with ANN models surpassing 98% accuracy. This indicates a powerful potential for these technologies in ensuring the integrity of honey products. Additionally, our study addresses both binary classification (pure vs. adulterated honey) and multi-class classification (various levels of adulteration), providing comprehensive insights into the detection process.

This research offers valuable contributions to the field of food safety by proposing a reliable and efficient approach to combat honey fraud. The integration of machine learning with hyperspectral imaging represents a promising advancement in quality assurance for the honey industry, ultimately protecting consumers and maintaining market integrity.

## Introduction

Food safety is crucial for public health and social security, especially in underdeveloped countries. Frequent incidents can reduce consumer confidence, disrupt market order, and cause social problems. Research on food safety has gained significant attention. Fruit production has grown dramatically over 57 years, with an average increase of about 240 million tons and a relative increase of about 40.3%. Fruit selection, transportation, and quality control have become important issues [1]. Fresh fruits, which contain essential minerals, vitamins, and dietary fibers, are essential for human diets. Identifying an efficient and nondestructive method to identify fruit quality has become a hot research direction.

Adulteration is the fraudulent addition of another substance to a substance with the intent to increase sales or profits. This can result in the degradation of food, medicines, and other items due to the inclusion of low-quality and cheap substances [2]. Impure substances are created by introducing impurities into a material or by removing a critical component from a material.

Adulterants lower the quality of food items while increasing the quantity available for consumption. In the food industry, adulteration is defined as adding an adulterant to a food item after processing. The purpose of adding an adulterant is unclear, but it is usually intentional to increase profit margins at the expense of public or customer health [3].

It is possible to tamper with honey, a popular sugar substitute with health benefits, by adding inexpensive sweeteners such as sucrose syrups, inverted syrups, high fructose corn syrups, or high fructose inulin syrups. The natural components of honey and adulterants may not be distinguished by standard detection methods, which could alter the chemical and physical properties of the honey.

Due to its many health advantages, such as its antimicrobial, anti-inflammatory, and anti-carcinogenic qualities, honey is consumed widely. Because of its high nutritional content and unique flavors, which draw counterfeiters, authenticity is essential.

Techniques such as Melissopalynology [4], which identifies adulterated honey, can be used to determine its botanical origin or maker compounds. The higher demand and limited availability of honey have led to different forms of honey adulteration, either directly by adding syrups to natural honey or indirectly by feeding honey bees with sugar syrups [5]. A need for reliable and cost-effective quality control methods to detect honey adulteration is emerging to ensure the safety and quality of honey.

In recent years, the problem of honey adulteration has become more serious. It has seriously damaged the interests of both producers and consumers. Therefore, it is urgent to develop an efficient and accurate honey counterfeit detection technology [6]. The raw materials of these carbohydrates are not only easy to obtain but also adulterated with these carbohydrates, the taste of honey is significantly improved, and it is difficult to detect. These reasons have caused honey to be an easy target of adulterators for economic gains [6,7]. Honey quality detection is crucial for ensuring food safety and improving food circulation efficiency. Traditional methods, such as instrumentation, manual labor, and manual labor, are time-consuming and labor-intensive. The review of consumer food safety literature reveals several gaps that could potentially contribute to the development of food-borne diseases in the home setting.

Machine Learning (ML) technologies have been successfully applied in various fields, such as image identification, voice identification, natural language interpretation and translation, and food safety challenges. Artificial Neural Networks (ANN), Support-vector machines (SVM) K Nearest Neighbors, Random Forests, and Decision trees are some of the ML technologies that can be used to classify honey samples. Therefore, this research presents a comparative study of these machine-learning algorithms.

### Related works

Nowadays, machine learning (ML) is used in developing adaptive intelligent systems that can perform complex tasks that are beyond human abilities. Some of the areas of applications of ML algorithms include pattern recognition, image processing, natural language processing, and medical diagnostics, to mention just a few [8]. Different types of ML algorithms exist, in the context of how they are applied to the field of adulteration detection. There are some technologies available to classify honey, but most of these methods are destructive, and too costly in terms of manpower, money, and time [9]. Here, destruction means that the conventional technologies would damage or compromise the honey samples. This study focuses on improving the detection of honey adulteration using machine learning by comparing the popular algorithms. In this section, the review of related research works is discussed as follows.

Oroian et al. [10] studied honey adulteration with fructose, glucose, and hydrolyzed inulin syrup and reported that it influenced some physicochemical properties such as pH, electrical conductivity, and water activity. Guler et al. [11] investigated changes in viscosity for adulterated honey and reported an increase in viscosity with a sugar syrup concentration increase. Several methods were used to evaluate direct adulteration in honey. Kelly et al. [12] reported the use of near-infrared transfectant spectroscopy to detect Irish honey adulteration by high fructose corn syrup and beet inverted syrup. Gallardo-Velazquez et al. [13] investigated the use of mid-infrared Fourier transform spectroscopy to quantify the content of honey adulterants including HFCS, corn syrup, and inverted sugar. RuizMatute et al. [14] reported the use of GC-MS for the detection of honey adulteration with high fructose Inulin syrups. Liquid chromatography (LC) and gas chromatography (GC) have been used simultaneously to detect exogenous sugars in honey by appropriate fingerprints of adulteration. Kumaravelu and Gopal [15] reported the use of near-infrared spectroscopy and partial least square regression for the detection and quantification of four honey types adulteration by jaggary.

Siddiqui et al. [16] present A thorough analysis of honey adulteration methods for the years 2000–2016. According to the authors, NMR spectroscopy is a potent technique for identifying genuine honey and detecting adulteration by different types of sugar.

To confirm the botanical provenance and authenticity of honey, many studies present recent developments in data mining, machine learning, and multivariate data analysis techniques. Numerous analytical techniques are covered, such as those by Wu et al. [17], Cuevas-Glory et al. [18], and Trifković et al. [19]. In addition, the study determines which parameters are most commonly used in conjunction with these methods to determine the location and floral origin of honey.

The research stated in [20] aims to identify various types of milk adulterants, detection methods, and health hazards associated with milk product adulteration. The proposed project investigates the fractional-order element-based impedance sensor for its potential use in detecting milk adulteration. The low-cost, user-friendly instrumentation system is expected to be commercialized soon. An automated sensing system for synthetic milk detection, based on a microcontroller, has been created to reduce reliance on specialized labor and improve efficiency. The dipole layer capacitance at the interface of the impedance sensor and contaminated milk is considered during the modeling process. An electrical equivalent circuit is built, and the detection of milk adulteration is classified using Recurrent Neural Networks. The proposed work is estimated to have an accuracy rate of 92.31 percent, a sensitivity rate of 75.23 percent, and a specificity rate of 90.12 percent, all higher than the current rate [20].

The recent work stated in [21] proposed an efficient and nondestructive method for detecting fruit freshness using the machine learning algorithm convolutional neural network (CNN). Traditional methods, such as instrumentation, testing reagents, or manual labor, are time-consuming and labor-intensive. Fruit, a high-value food, is susceptible to spoilage during packaging, transportation, and sales. The paper shows that CNNs have good performance in identifying fruit freshness and discusses the overfitting of machine learning based on experimental results.

The majority of the studies aim for the determination of the botanical origin of honey, with only a few aiming for geographical origin. A possible reason for the extra effort by researchers to ascertain the botanical origin is that it causes a greater impact on the composition and properties of honey.

Several studies have used Fourier transform infrared spectroscopy to detect adulteration in honey with cane sugar. The accuracy of predicting sugar concentration was 93.75% for one type of honey but below 80% for three different honey types. This shows that spectroscopy can be used with machine learning techniques to predict adulteration in honey, but the ability to predict sugar concentration across a range of honey must be improved. Hyperspectral imaging is a promising tool for food quality assurance, allowing spatial information to be used alongside spectral information. It has been used for various food quality applications, including determining different properties of grapes and predicting the properties of chocolate. The problem needs further investigation by evaluating the effect of feeding bees at different sugar concentrations and evaluating the resulting physiochemical properties of the honey. It is also necessary to develop a new reliable and cost-effective method for detecting adulteration in honey. Therefore, the objective of this study is to use the outperformed algorithm from the comparative analysis among K-nearest neighbors, Support vector machine, and random forest. Decision tree and ANNs to classify and predict. Compared to existing approaches, our proposed model relies on hyper-spectrum images. Moreover, our approach shows the discovery to overcome generalization to unknown honey types problems and improve the accuracy of existing models using hyperparameter tuning with enhanced performance.

## Materials and methods

The study used hyperspectral imaging technology to collect segmented and pre-processed hyperspectral images of adulterated honey samples. The images underwent preprocessing to enhance quality and remove noise. Relevant features were extracted from each image, serving as input variables for machine learning models. Five machine learning algorithms were selected: Artificial Neural Networks (ANNs), Support Vector Machines (SVMs), Random Forests, K-Nearest Neighbors (KNN) and Decision Tree. The models were trained using the extracted features, learning to classify honey samples as pure or adulterated based on their spectral characteristics. Performance was evaluated using cross-validation techniques, assessing accuracy, precision, recall, and F1-score. A comparative analysis was conducted to identify the most effective approach for detecting honey adulteration.

**Fig 1:**
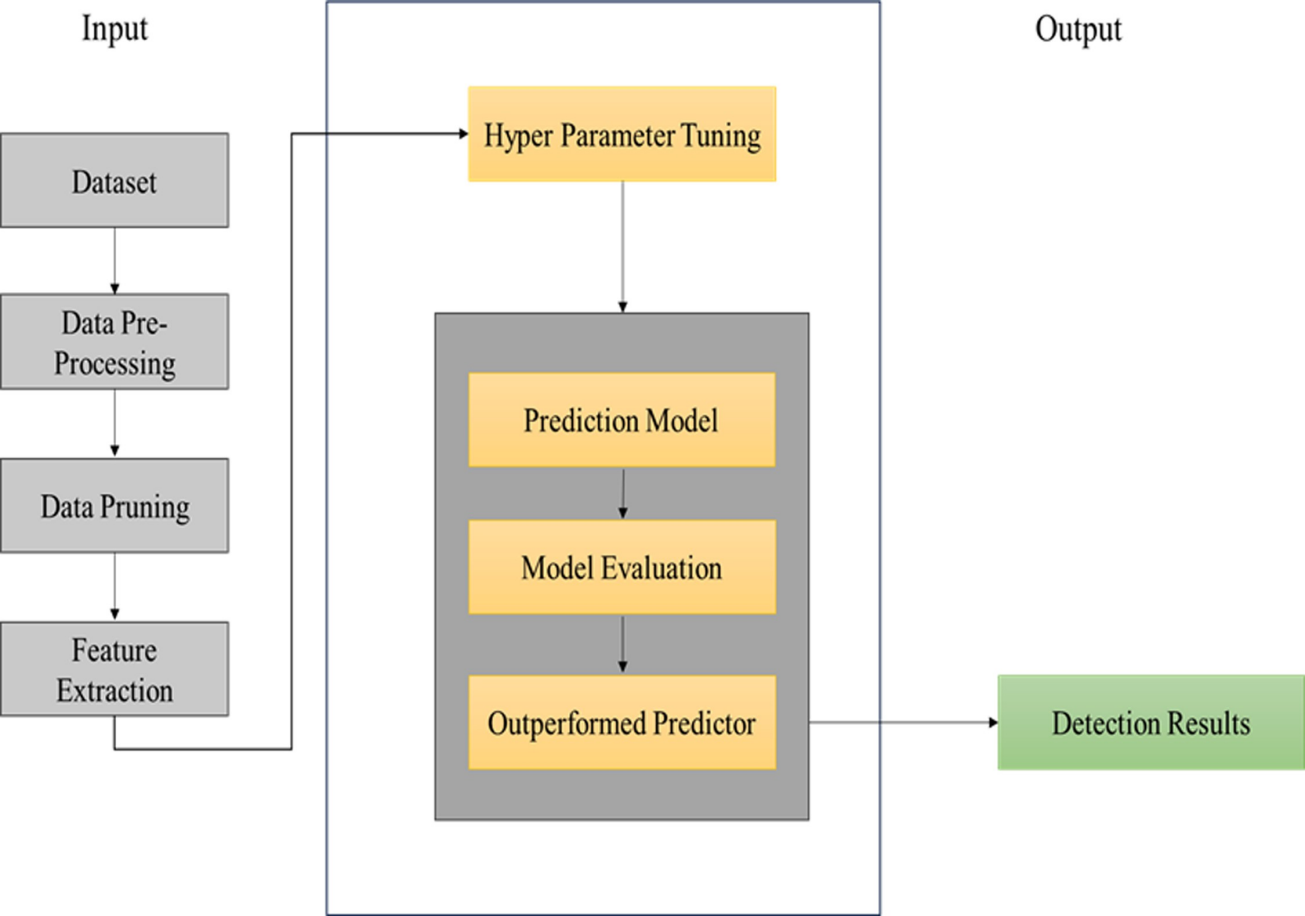
The architecture of the proposed Solution.

The methodology involved a systematic approach to leveraging machine learning techniques for honey adulteration detection, ensuring reliability and effectiveness in addressing research objectives. Fig 1 illustrates the procedure we follow in conducting this research.

### Dataset

The dataset used in this study consists of segmented and pre-processed hyperspectral images of adulterated honey samples. Hyperspectral imaging is a non-destructive analytical technique that combines conventional imaging with spectroscopy to capture both spatial and spectral information from an object. In the context of honey adulteration detection, hyperspectral imaging allows for the acquisition of detailed spectral data across a range of wavelengths, enabling the identification of adulterants based on their unique spectral signatures.

**Fig 2:**
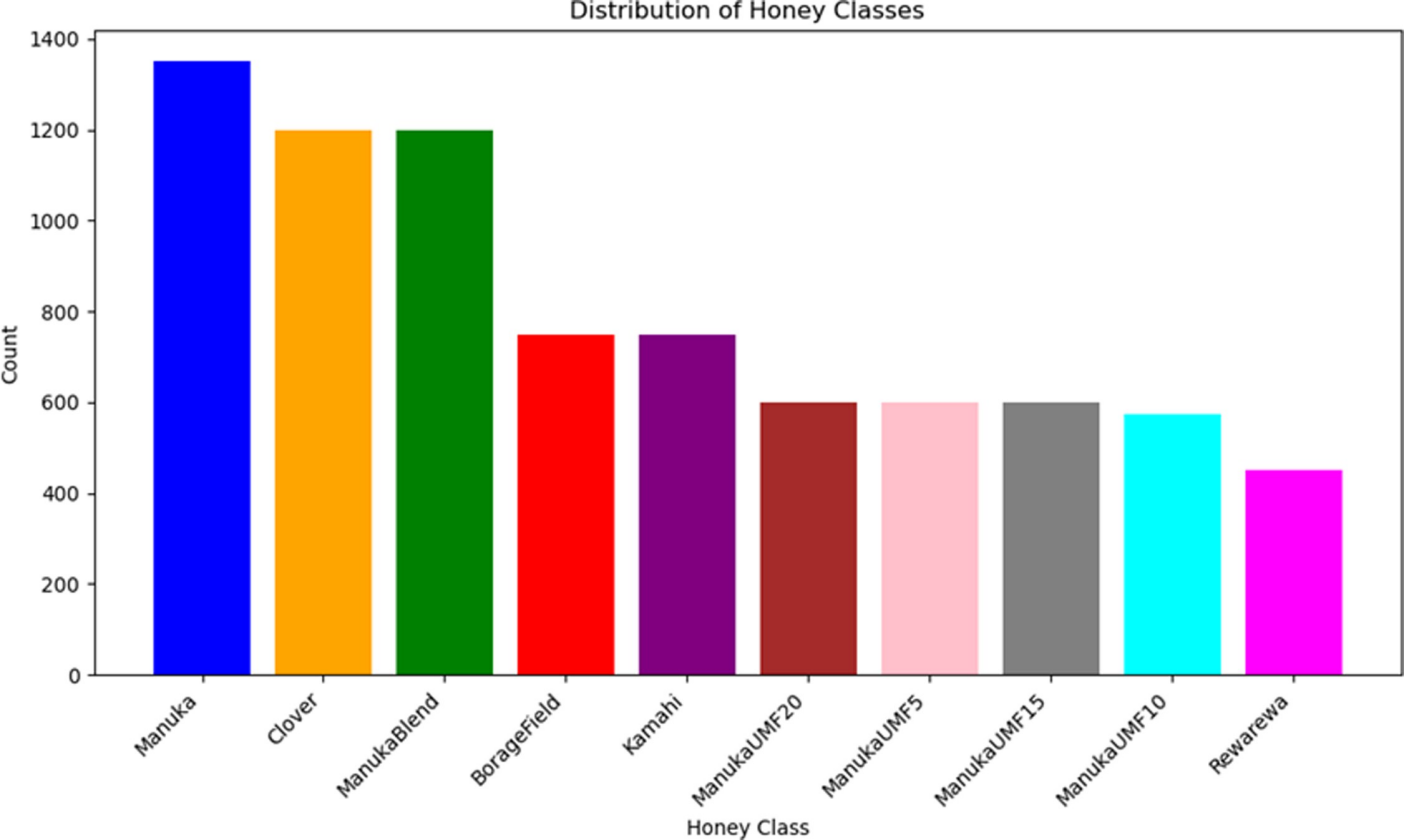
Class Distribution.

Overall, the dataset comprises 12 different honey products from seven different brands with 12 different botanical origins labels as shown in Fig 2. Half of the samples are types of Manuka honey which is a premium NZ honey type, and the other half are various types of other NZ honey. ManukaUMF5 refers to a specific type or grade of Manuka honey with a UMF (Unique Manuka Factor) rating of 5.

UMF is a grading system used to measure the potency and quality of Manuka honey, with higher UMF ratings indicating higher levels of beneficial compounds.

Table 1 shows the makeup of the dataset from these different kinds of honey. In creating the dataset, we sampled and captured images of all the honey at each sugar concentration; however, some mixtures were of low quality, and we could not include the images in the final dataset. The full dataset of honey samples contains 8675 total instances.

**Table 1:** The top 10 Classes with the most entries in the dataset.

Honey Class	Count
Manuka	1350
Clover	1200
ManukaBlend	1200
BorageField	750
Kamahi	750
ManukaUMF20	600
ManukaUMF5	600
ManukaUMF15	600
ManukaUMF10	575
Rewarewa	450

Each instance is the result of spatial segmentation of the hyperspectral imagery. It contains 128 features ‐ representing the spectral wavelengths of the hyperspectral camera.

Table 1 represents the number of training and testing examples available. Each sample contains 25 examples following segmentation.

**Fig 3:**
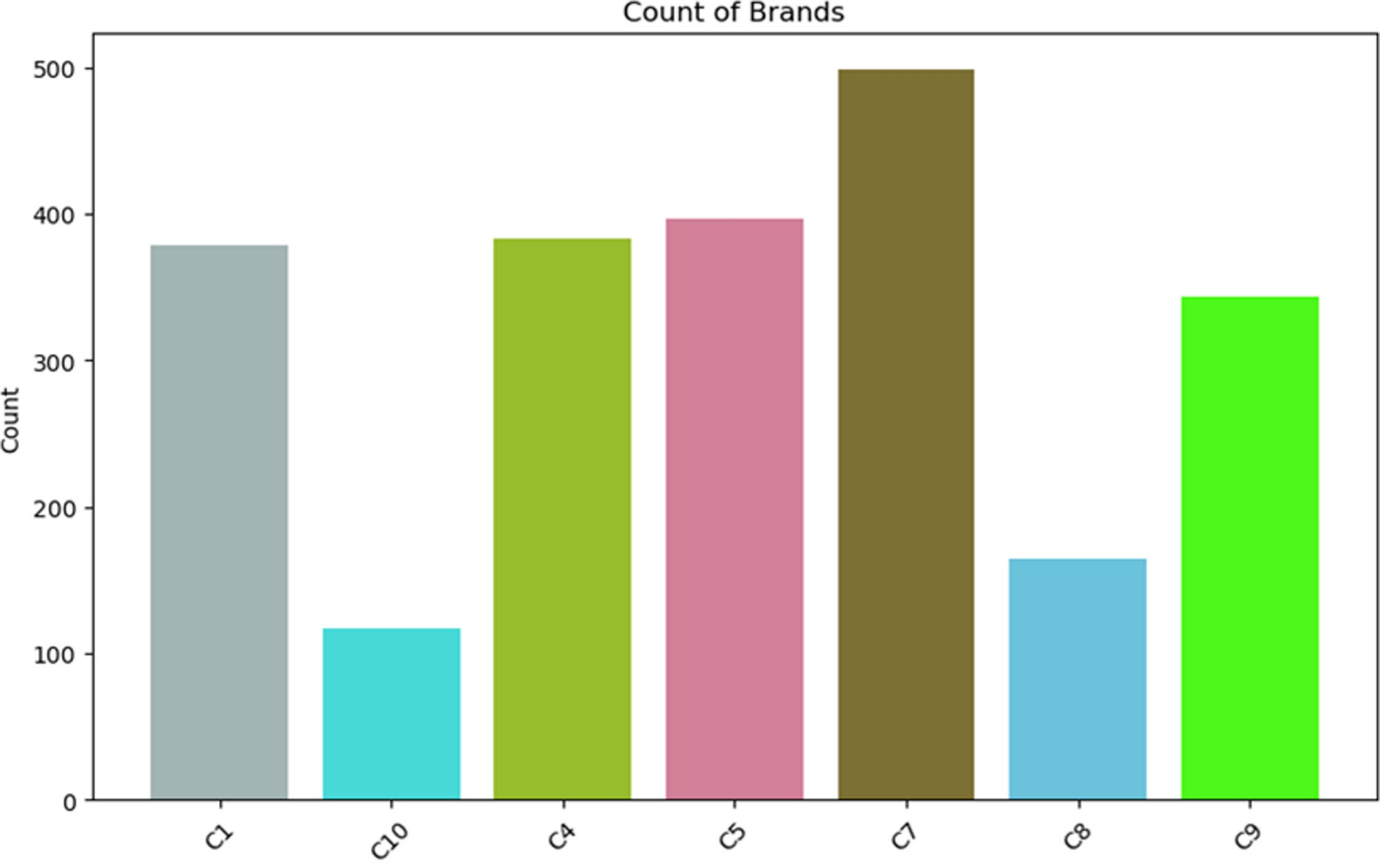
Distribution of Brand.

Fig 3 shows the brand distribution along counts of the dataset. As the inspection graph indicates, the brand distribution has variety. Some brand has more count scores while some other brands have less.

### Data pre-processing

Before applying machine learning algorithms, the dataset undergoes pre-processing steps to enhance its quality and suitability for analysis. This includes normalization of spectral data to ensure consistent scale across features, segmentation of hyperspectral images into training examples, and removal of low-quality samples. The pre-processing aims to standardize the data and improve the performance of subsequent machine learning models.

The pre-processing step follows the segmentation and uses a simple normalization approach, where the mean of the spectra is forced to be at 0, and the standard deviation to be 1. Other normalization approaches were also considered; however, this was experimentally found to be the best approach for this dataset.

The hyperspectral imaging system first calibrates using a dynamic white reference technique, and then it performs preprocessing segmentation. Using this technique, each hyperspectral image is divided into a five-by-five grid, yielding twenty-five training examples. The segments are averaged to represent the sample of honey as a whole, and the final data set is normalized before machine learning techniques are applied.

**Fig 4:**
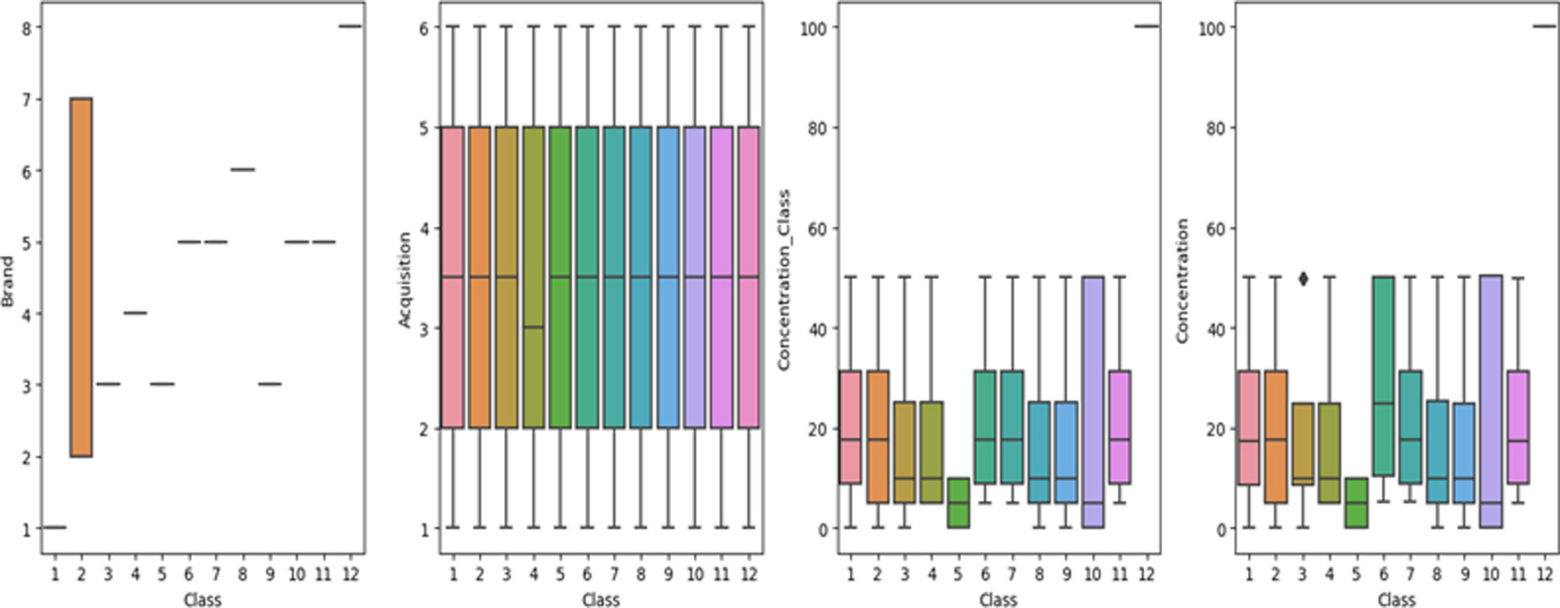
Outlier detection.

Outlier detection is a crucial step in identifying parameters affected by outlier tools from thousands of parameters. It involves separating log files based on recipe and tool numbers, processing them to calculate means and standard deviations using MapReduce, and performing outlier detection. An outlier detection technique (ODT) is used to detect anomalous observations that do not fit the typical statistical distribution of a dataset. In this study, simple methods that use statistical tools like boxplots have been employed to represent the distributions of samples corresponding to each feature. Fig 4 likely presents boxplots for each feature in the dataset, providing a visual representation of the distribution of values and identifying potential outliers. Fig 4 provides valuable insights into the distribution of features in the dataset and highlights potential outliers that may require further investigation. By addressing outliers and understanding the characteristics of each feature, researchers can enhance the effectiveness of their machine-learning algorithms for detecting honey adulteration. Understanding the distribution of features is essential for developing accurate machine-learning models for honey adulteration detection. Identifying outliers helps ensure that the models are trained on high-quality data, leading to more reliable predictions.

### Data pruning

We classify the botanical origins of honey using the most promising methods currently available, and we also take into account the theory of adulteration from related applications. By lowering the problem’s complexity, feature reduction can lessen overfitting and enhance performance. Principal component analysis (PCA), feature ranking-based feature selection of the k best features, and autoencoder techniques are the feature reduction techniques employed. One common technique for extracting and reducing features is PCA. A set of orthogonal vectors is formed when the features are combined linearly. This approach retains only the features with high variance.

### Feature extraction

Feature extraction involves identifying and selecting relevant information from the dataset to represent each honey sample. In this study, spectral features extracted from hyperspectral images serve as input variables for the machine learning models. Additionally, attributes such as brand, acquisition, and concentration class are considered for classification and prediction tasks.

The primary characteristics of hyperspectral images are the camera’s operating wavelengths, which are utilized to analyze honey. To test the algorithms’ generalization ability and divide the dataset into training and testing sets, additional attributes are taken into account. All honey types within a brand can be categorized using the ’Brand’ attribute, which represents the honey’s manufacturer. The ’Acquisition’ attribute denotes the various image samplings for identical honey types and brands. Six samples are taken, numbered one through six, for every distinct honey jar. As a result, instances from a segment that is part of the training set are avoided and a balanced distribution of all honey types is obtained. The botanical class of honey is indicated by the ’Class’ attribute.

### Hyper parameters tuning

The model parameters are enhanced or tuned by the training process. We run data through the operations of the model, compare the resulting prediction with the actual value for each data instance, evaluate the accuracy, and adjust until to get the best values. Hyperparameters are tuned by running the whole training data to look at the aggregate accuracy and adjust. Dataset pruning is the process of removing sub-optimal tuples from a dataset to improve the learning of a machine learning model. The idea of pruning is to consider a subset of hyperparameter configuration space to avoid unnecessary functions or attributes of data.

### Prediction model

Various machine learning algorithms are employed for classification and prediction tasks, including Artificial Neural Networks (ANN), Support Vector Machines (SVM), K-nearest neighbors (KNN), Random Forests (RF), and Decision Trees (DT). Each algorithm is trained on the pre-processed dataset using appropriate hyperparameters to optimize performance. The modeling process consists of selecting models that are based on various machine learning techniques used in the experimentation. The model selection process for detecting honey adulteration using hyperspectral imaging data involves choosing machine learning algorithms based on their suitability for handling high-dimensional data, learning complex patterns, and achieving accurate classification. The selected algorithms include Artificial Neural Networks (ANN), Support Vector Machines (SVM), K-nearest neighbors (KNN), Random Forests (RF), and Decision Trees (DT).

Artificial Neural Networks (ANN): Chosen for its ability to learn complex patterns from large datasets, making it suitable for image classification tasks. It learns to distinguish between genuine and adulterated honey samples based on their spectral signatures extracted from hyperspectral images.

Support Vector Machines (SVM): Selected for its capability to separate classes by finding the optimal hyperplane with maximum margin. It’s effective for binary classification tasks and classifies honey samples as pure or adulterated based on their spectral features.

K-Nearest Neighbors (KNN): Chosen for its simplicity and effectiveness in handling non-linear relationships in data. It assigns the majority class among the nearest neighbors of each sample in the feature space, allowing for accurate classification of honey samples.

Random Forests (RF): Selected for its robustness and ability to handle noisy and high-dimensional data. It combines multiple decision trees to improve classification accuracy by learning from different subsets of features.

Decision Trees (DT): Chosen for their simplicity and interpretability, making them suitable for understanding the decision-making process behind classification tasks. They partition the feature space based on spectral characteristics to accurately classify honey samples.

Overall, these machine learning algorithms are applied to develop accurate and robust classification models for detecting honey adulteration, ensuring food safety and quality. The goal is to identify the best classifier for the analyzed problem. Each classifier must therefore be trained on the featured set and the classifier with the best classification results is used for prediction. The details of the algorithms used in this study are discussed as follows.

### Artificial Neural Network (ANN)

Artificial neural networks (ANNs) have gained popularity for classification, clustering, pattern recognition, and prediction in various disciplines, including artificial intelligence, information security, big data, cloud computing, internet, and forensic science [22]. ANNs’ full applications can be evaluated based on factors such as accuracy, processing speed, latency, performance, fault tolerance, volume, scalability, and convergence. ANNs are particularly useful for image recognition and natural language processing due to their self-learning, adaptivity, fault tolerance, and advancement in input-to-output mapping. ANNs are effective in handling complex and non-complex problems in various spheres of life, including agriculture, science, medical science, education, finance, management, security, engineering, trading commodities, and art [23]. However, there is a growing need to adopt a systematic approach in the development phase to improve performance, such as addressing factors such as data set choice, data accuracy, data instrument, data standardization, data inputs, data division, preprocessing, validations, processing, and output techniques [24].

In this study, Artificial Neural Networks (ANNs) were employed as a machine learning technique to classify and predict adulteration in honey samples using hyperspectral imaging data. ANNs are a type of deep learning algorithm inspired by the structure and function of the human brain’s neural networks. They consist of interconnected nodes organized in layers: an input layer, one or more hidden layers, and an output layer.

For this study, ANNs were trained on segmented and pre-processed hyperspectral images of adulterated honey samples. Each pixel in the hyperspectral images represents a specific wavelength of light reflected by the honey sample, providing detailed spectral information. The ANN model was designed to learn patterns and relationships within these spectral data to accurately classify honey samples as either pure or adulterated, as well as to predict the degree of adulteration.

During training, the ANN adjusts its internal parameters through a process called backpropagation, where errors between predicted and actual outcomes are used to update the connections between nodes. This iterative process continues until the model achieves satisfactory performance on a validation dataset.

The performance of the ANN was evaluated based on metrics such as precision, recall, F1-score, and accuracy. Precision represents the proportion of correctly classified adulterated honey samples among all samples classified as adulterated, while recall measures the proportion of correctly classified adulterated honey samples among all actual adulterated samples. The F1-score is the harmonic mean of precision and recall, providing a balance between the two metrics. Accuracy, on the other hand, represents the overall correctness of the classification results.

ANNs were utilized in the study to leverage the spectral information captured by hyperspectral imaging for the accurate detection and quantification of honey adulteration. Through the training and evaluation of ANN models, the study aimed to develop a reliable and efficient tool for ensuring the authenticity and safety of honey products.

### Support Vector Machine (SVM)

The support vector machine approach is based on the notion of estimating (maximum) margins. The algorithm aims to discover a hyperplane (decision boundary) that is placed between the data points of the different classes and is as far away from the data points as possible. The support vectors are the data points nearest to the hyperplane [25]. Because the support vectors influence the position and orientation of the hyperplane, they are utilized to maximize the margin of the classifier. The hyperplanes (H) are defined by giving weights (w) to each feature as well as some bias (b), the combination of which predicts the target variable (y).


w*xi+b≥+1whenyi=+1

w*xi+b≥−1whenyi=−1

The bias term ensures that the separating hyperplane does not have to go through the origin. The weights are proportional to the feature’s importance. The features most important for splitting the data do have higher weights. When different classes are not linearly separable, the support vector machine uses a technique called ‘the kernel trick’. The basic idea of the support vector machine kernel is that the function transforms a low-dimensional input space into a higher-dimensional space, to be able to separate the target classes with a hyperplane. Different kernels can be specified, such as the linear kernel, the polynomial kernel, the radial basis function kernel, and the sigmoid kernel. Since a thorough explanation of the different kernels is beyond the scope of this thesis, this is not discussed here.

The primary reason for using the support vector machine is the ability of the algorithm to capture complex relationships without applying transformations to the data. By projecting the data into a higher-dimensional space, the support vector machine can model non-linear patterns in the data [26].

For this study, SVMs were trained on segmented and pre-processed hyperspectral images of adulterated honey samples, similar to the approach used for Artificial Neural Networks (ANNs). Each pixel in the hyperspectral images represents a specific wavelength of light reflected by the honey sample, providing detailed spectral information. The SVM model was designed to learn patterns and relationships within these spectral data to accurately classify honey samples as either pure or adulterated.

The key concept behind SVMs is to find the optimal hyperplane that best separates the different classes in the feature space. In the case of binary classification (pure vs. adulterated honey), this hyperplane acts as a decision boundary that maximizes the margin between the classes, thereby enhancing the model’s generalization ability [27].

During training, the SVM algorithm determines the optimal hyperplane by identifying a subset of training samples called support vectors, which lie closest to the decision boundary. The model aims to maximize the margin between these support vectors while minimizing classification errors.

### K-Nearest Neighbors (KNN)

K-nearest-neighbor (KNN) classification is a fundamental and simple method used in classification studies when data distribution is unknown or difficult to determine. It is based on the idea that the nearest patterns to a target pattern provide useful label information. KNN assigns the class label to the majority of K-nearest patterns in the data space [28].

The KNN algorithms classify new data based on the class of the k nearest neighbors. The distance from neighbors can be computed using many distance metrics, such as Euclidean distance, Manhattan distance, Minkowski distance, and so on. In this study, Manhattan distance was used to compute the distance. The new data class may be determined by a majority vote or inverse proportion to the calculated distance. KNN is a non-generalizing approach since the algorithm stores all of its training data in memory, which may be translated into a fast-indexing structure such as a ball tree or a KD tree. For this research, KNN was trained on pre-processed hyperspectral images of adulterated honey samples. Each pixel in these images represents a specific wavelength of light reflected by the honey, providing detailed spectral information.

During training, the KNN algorithm stores all available cases and their class labels. When presented with a new data point, it calculates the distances to all other points in the training set and identifies the k nearest neighbors.

Classification is then determined by the majority class label among the k neighbors, with the most frequent label assigned to the new data point. The choice of the parameter k is critical, influencing the model’s performance and generalization ability.

### Random forest

L. Breiman [29] presented the random forest algorithm, and it has proven to be an effective technique for regression and classification. It combines several randomized decision trees and averages the results to get an overall prediction. This approach is flexible, appropriate for solving complex issues, and appropriate for a range of learning assignments. With an emphasis on mathematical forces, parameter selection, resampling mechanisms, and variable importance measures, this article examines recent theoretical and methodological advancements for random forests [29].

Random Forest is a mast er learning technique used for grading and regression. It’s a kind of ensemble technique that combines a bunch of weak models to create a powerful model. The random forest generates several tresses. Voting for that class should be used to categorize each tree. This is a categorization. The forest chooses the classification with the most votes. Take the test to evaluate the features and choose trees to predict and save the findings [30].

In the study focused on detecting honey adulteration using hyperspectral imaging, the Random Forest algorithm played a key role. Random Forest is an ensemble learning method utilized for classification tasks. In this context, it was applied to analyze hyperspectral images of honey samples, where each pixel represents a specific wavelength of reflected light. These images provide detailed spectral information crucial for identifying adulteration. During training, Random Forest constructs numerous decision trees based on random subsets of the training data and features. This randomness helps mitigate overfitting and enhances the model’s ability to generalize to new data. In the context of honey adulteration detection, Random Forest aggregates the predictions of individual decision trees to classify samples.

By considering spectral patterns across multiple wavelengths, it accurately discerns between pure and adulterated honey.

### Decision tree

Decision tree classifiers are widely used in many domains, such as text classification, user smartphone classification, images, and medical disease analysis. A decision tree is a graph that resembles a tree, with internal nodes standing in for attribute tests, branches for test results, and leaf nodes for class labels. The process of choosing a path from the root node to the leaf forms the classification rules. Each intermediate node’s input data is analyzed by identifying attributes and the values that correspond with them to construct the tree [31]. Depending on the test conditions at each node along the path, it can prefigure new data by going through all of the internal nodes. By evaluating vast amounts of intrusion detection data, decision trees can enhance real-time security systems and detect noteworthy network features that point to malicious activity. They offer a comprehensive set of guidelines that are simple to comprehend and work well with real-time technologies. It uses real data-mining algorithms to help with classification. A decision-tree process will generate the rules followed in a process [32]. Decision trees are useful for helping you choose among several courses of action and enable you to explore the possible outcomes for various options to assess the risks and rewards for each potential course of action. These decisions generate rules, which then are used to classify data [33]. Decision trees are the favored technique for building understandable models.

In the realm of honey adulteration detection using hyperspectral imaging, the Decision Tree algorithm serves as a fundamental tool. This method operates by recursively partitioning the dataset based on different spectral features at each node of the tree. The decision-making process involves evaluating specific wavelengths captured by the hyperspectral imaging system, expressed in nanometers (nm).

In the context of our study, the Decision Tree algorithm analyzes the hyperspectral data, focusing on the wavelengths denoted by labels such as Anm, Bnm, Cnm, Dnm, Enm, Fnm, and Gnm. These labels represent the specific nanometer values corresponding to the wavelengths at which spectral data were collected. By assessing the spectral characteristics associated with different honey samples, the Decision Tree algorithm builds a hierarchical structure that facilitates the classification of samples into distinct categories.

The decision nodes in the tree represent specific wavelength bands, and the branches emanating from each node signify the outcomes of the decision based on the observed spectral information. As the algorithm traverses the tree, it ultimately leads to leaf nodes, each corresponding to a classification decision regarding the presence or absence of honey adulteration.

By leveraging the Decision Tree algorithm, our research aims to determine the crucial spectral features indicative of adulteration, contributing to the development of an effective and interpretable model for honey quality assurance.

### Performance measures

To accurately detect adulterated honey using various algorithms, proper evaluation needs to be conducted. This paragraph will discuss the various evaluation criteria used to compare the different algorithms. First, the data will be partitioned into a training set and a testing set. A resampling procedure known as 5-fold cross-validation will then be used on the training set to prevent potential biases. All classifiers were validated using 5-fold cross-validation. Performance is measured based on precision, recall, and accuracy. Precision is the ratio of correctly predicted positive observations to the total predicted positive observations [34]. Recall is the ratio of correctly predicted positive observations to all observations in actual class [35]. The easiest performance metric to understand is accuracy, which is just the proportion of properly predicted observations to all observations [36]. In this study, performance is measured based on the following parameters as shown below in Eqs 1, 2, and 3.

$$Precision=\frac{TP}{TP+FP}$$ (1)

$$Recall=\frac{TP}{TP+FN}$$ (2)

$$Accuracy=\frac{\left(TP+TN\right)}{TP+TN+FP+FN}$$ (3)

$$F1\;Score=\frac{\left(2*\;Recall\;*\;Precision\;\right)}{\;Recall\;+\;Precision\;}$$ (4)

where TP  =  True Positive, TN  =  True Negative, FP  =  False Positive, and FN  =  False Negative.

### Results and discussion

This section describes the accuracy of the adopted models. The results of the decisions made in the prediction phase were collected, for each algorithm, in the relative confusion matrix. This is a matrix where the values predicted by the classifier are shown in the columns and the real values of each instance of the test-set are shown in rows. To proceed with the performance evaluation, we used the confusion matrix to derive a series of fundamental metrics to quantitatively express the efficiency of each algorithm: recording F1 Score, precision, and recall.

### Environmental setup

Python was selected for code development with tools like Notepad and Anaconda because of its simple syntax. The mean values were computed using NumPy and Pandas, and the model was assessed using Scikit-learn’s random forest and tree module. Machine learning tools such as classification, regression, clustering, dimensionality reduction, model selection, and pre-processing are included in Sclera, also referred to as sclera. In the study, well-known algorithms like ANN, SVM, KNN, random forests, and decision trees were employed. Packages for collections and surprises were also employed. The algorithms’ performance was assessed and compared using cross-validation techniques.

## Result

In this study, we evaluate the classification accuracy in two different scenarios: firstly, binary classification between pure honey and all concentrations of adulterated honey. Then, the classification of adulteration % into categories of 0%, 5%, 10%, 25%, and 50% is shown in Table 2. These two scenarios represent a realistic real-world need, which is to, most importantly, be able to detect if a honey sample is fraudulent.

**Table 2:** Overall adulterated honey data set from each brand and botanical origins label of honey.

Brand	Class	Adulteration				
		0%	5%	10%	0%	50%
C1	Clover	150	C1	Clover	150	C1
C10	MultiFloral	150	C10	MultiFloral	150	C10
	ManukaUMF5			ManukaUMF5		
	ManukaUMF15			ManukaUMF15		
	ManukaUMF20			ManukaUMF20		
C4	ManukaUMF10		C4	ManukaUMF10		C4
C5	ManukaBlend		C5	ManukaBlend		C5
C7	BorageField	150	C7	BorageField	150	C7
	Kamahi	150		Kamahi	150	
	Rewarewa	150		Rewarewa	150	
C8	ManukaBlend	150	C8	ManukaBlend	150	C8
C9	Manuka	150	C9	Manuka	150	C9

Once adulteration is detected, estimating how much adulterant is used would become crucial. Our concentration detection is limited to a classification problem with four possible adulteration concentrations due to the data being heavily skewed toward these four concentrations. This data skew was by design when we created the dataset, as we aimed to evaluate how well the classifiers could detect different ranges of concentrations.

In classification results, the classifier is trained to classify adulterated vs non-adulterated honey generally. The training and testing set consists of all the different concentrations of sugar adulteration we have captured as adulterated honey and the pure honey captured as non-adulterated. The classifiers used have been used in previous work on honey botanical origins classification.

**Fig 5:**
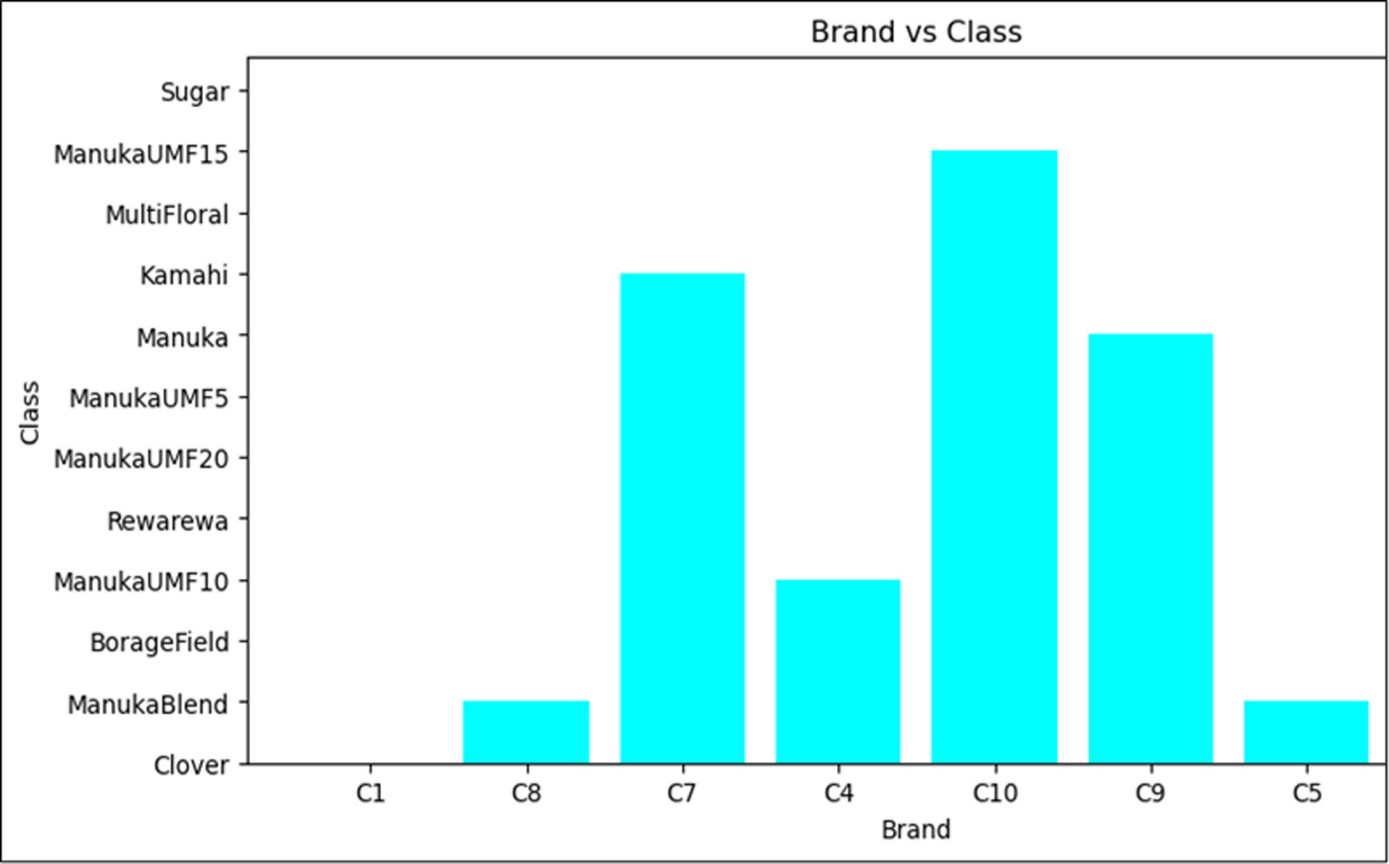
Bar chart of brand vs class distribution before preprocessing.

**Fig 6:**
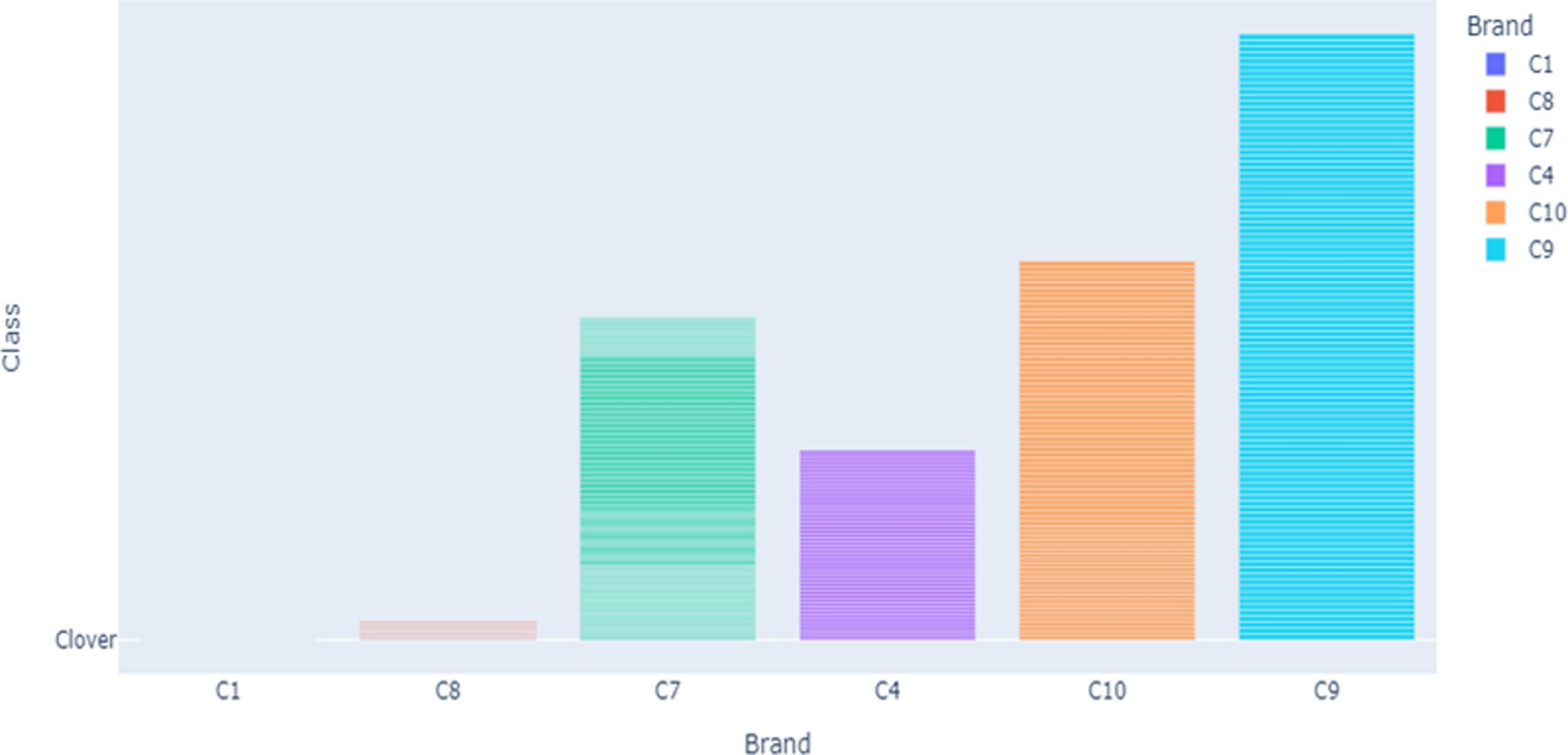
Brand vs class distribution after preprocessing.

**Fig 7:**
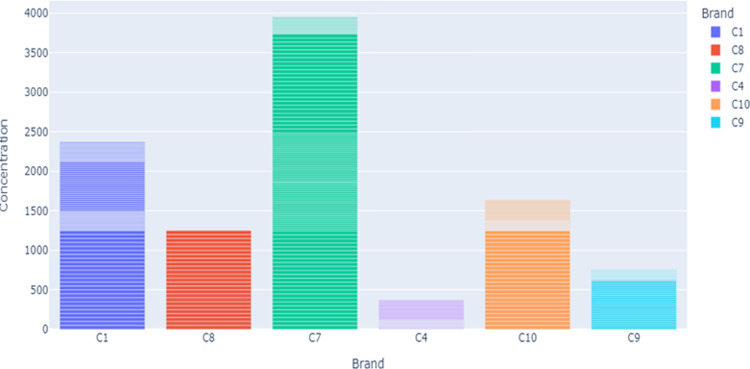
Concentration vs Brand.

Fig 5 shows the s distribution of brands along the class. As it is indicated, the brands C1, C5, and C8 have less count across the class compared to others. The researcher used this distribution for segmentation and normalization. Figs 6 and 7 present the brand with class distribution and Concentration Brand after preprocessing the pre-processing step performed.

Fig 6 depicts the relationship between brand and class distribution in the dataset.

The researcher analyses the data to magnify a sense of what additional work should be performed to quantify and extract insights from the data. The acquisition attribute represents the different sampling of images for the same type and brand of honey. As portrayed in Fig 7, for each unique brand of honey, there have been six samples taken and captured by the hyperspectral imaging system. Each image captured is numbered with an acquisition.

**Fig 8:**
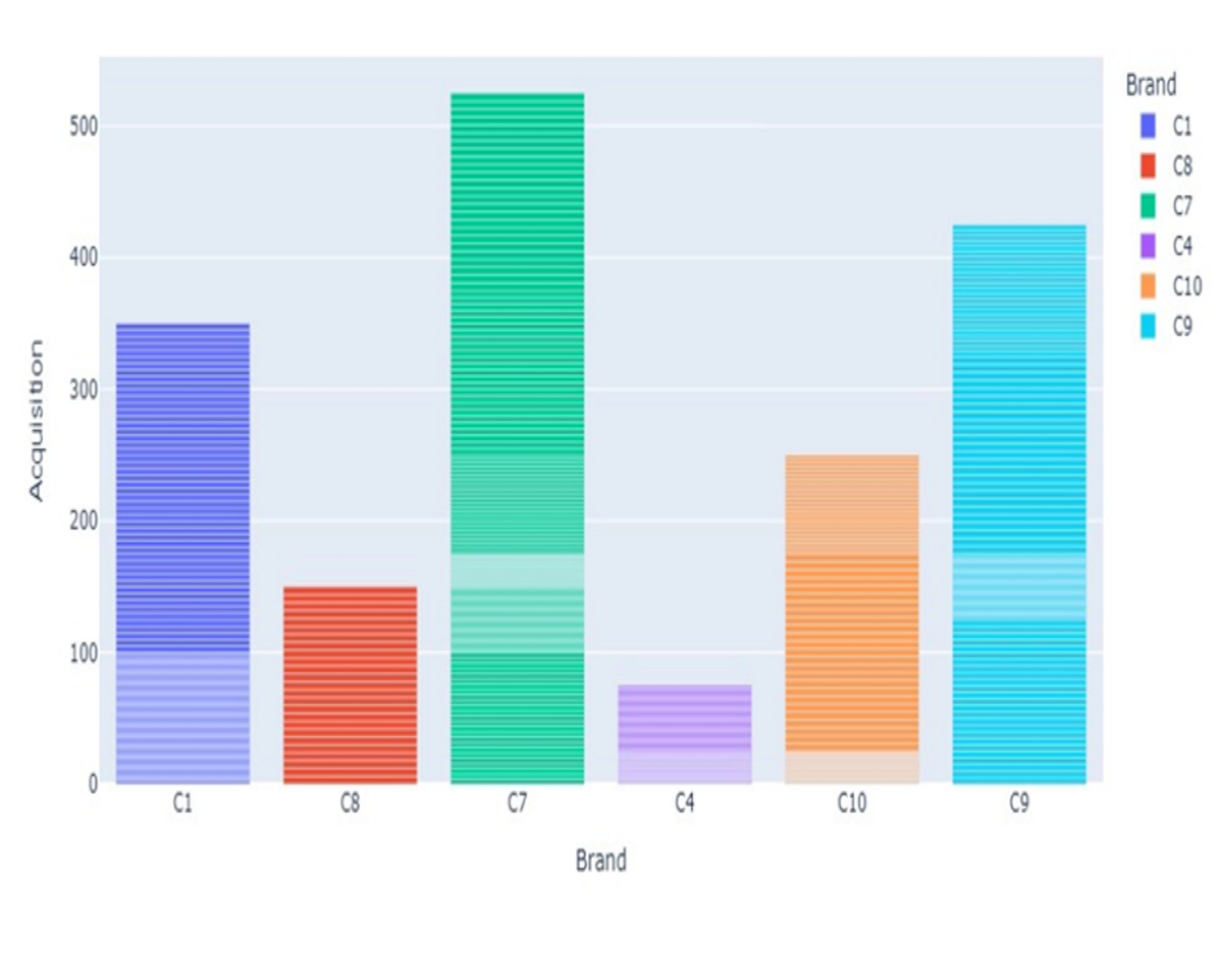
Distributions of Acquisition attribute along brands of honey.

Since the modeling step in this study uses random forest regression, there are two sugar concentration attributes. The first attribute is ‘concentration’ represents the actual concentration of sugar in the sample. The distribution of these attributes is presented in Fig 8.

This attribute is used for regression-type algorithms as it has the exact concentration of sugar that we have adulterated the pure honey sample with.

**Fig 9:**
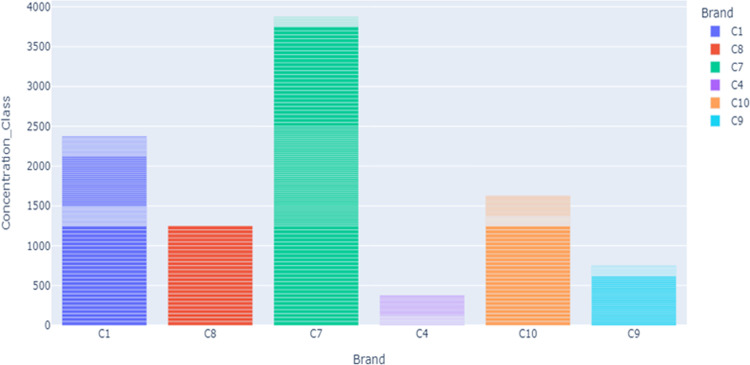
The distribution of the concentration_class.

The second attribute is ‘concentration_class’ this represents the class grouping of the adulterated sample. This attribute is used for classification problems to detect what group the adulterated honey should fit into. Fig 9 shows the distribution of the concentration_class.

The reduction in the number of brands resulted from correlation analysis conducted as part of the data preprocessing and feature selection process. By selecting brands that provide the most relevant and informative data points, we aimed to facilitate a deeper understanding of the factors influencing honey adulteration detection.

**Fig 10:**
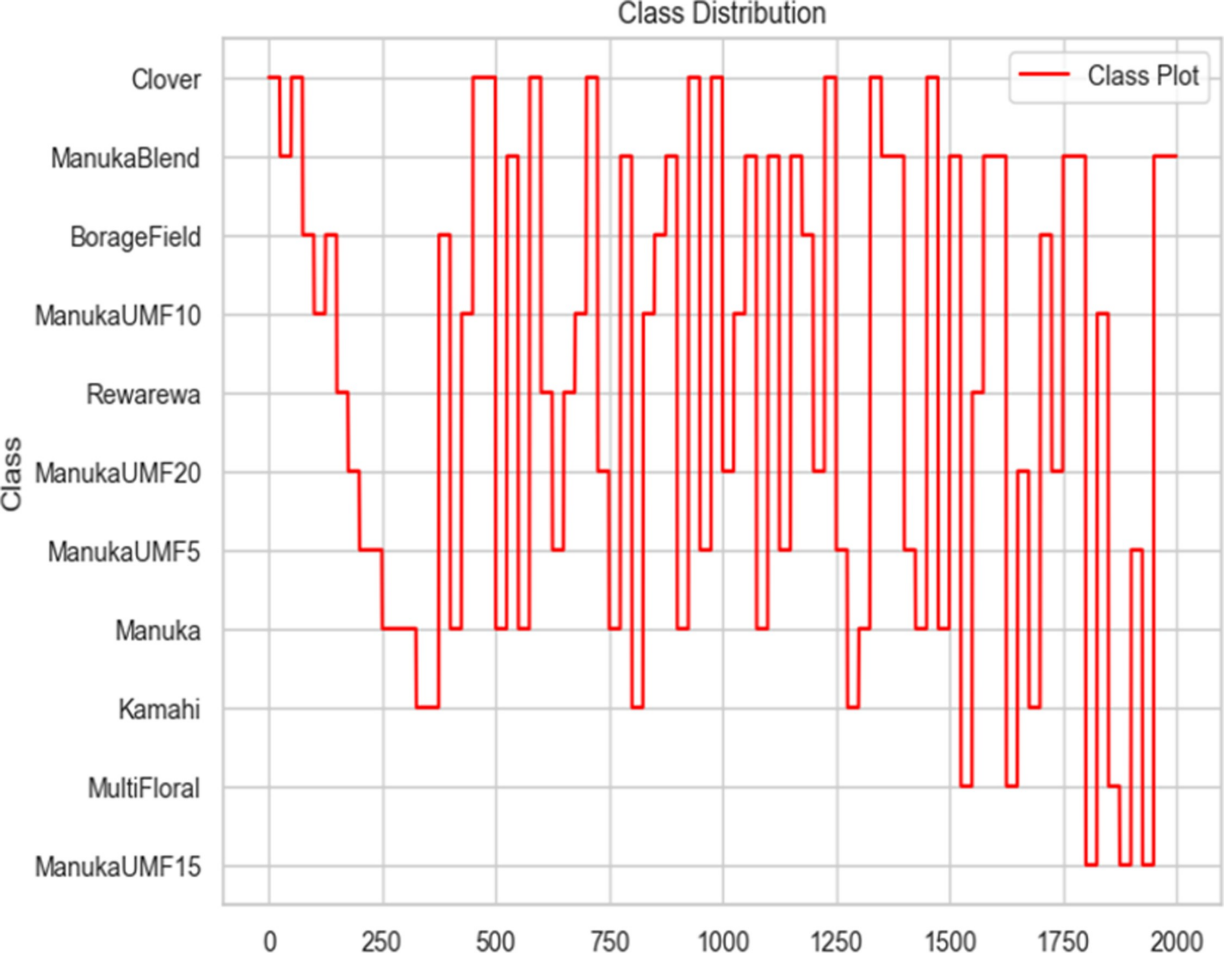
Distribution of Class Counts in the Dataset.

The class attribute indicates the class of honey, which is the botanical origin, and the UMF value if it is UMF-rated Manuka honey. Botanical origins have a huge impact on the value of honey, where some types are precious such as pure Manuka honey, and others are much more common and not considered valuable. Fig 10 shows the summary of the new class distribution of the preprocessed data. The visualization displays the distribution of classes in the dataset, represented by the "Class" variable. Each point on the line plot represents a different class, and the y-axis represents the count or frequency of each class in the dataset.

From the visualization, we can observe the distribution of classes across the dataset. Some classes may have higher counts, indicating a larger presence in the dataset, while others may have lower counts, indicating less representation. This information can be valuable for understanding the balance or imbalance of classes in the dataset, which can influence the performance of machine learning models trained on this data.

Researchers tried to improve the predictions of the model by enhancing some of the hyperparameters of the algorithm. To do this, the grid search cross-validation method was implemented. When a range of hyperparameter values is passed, grid search cross-validation automatically searches through the parameter space to find the best-performing set of hyperparameters. We used hyperparameter tuning for enhancement of the algorithm. Hyperparameter tuning is used for the automatic enhancement of the hyperparameters of a model. Hyperparameters are all the parameters of a model that are not updated during the learning and are used to configure the algorithm to lower the cost function of the learning rate for the gradient descent algorithm.

**Fig 11:**
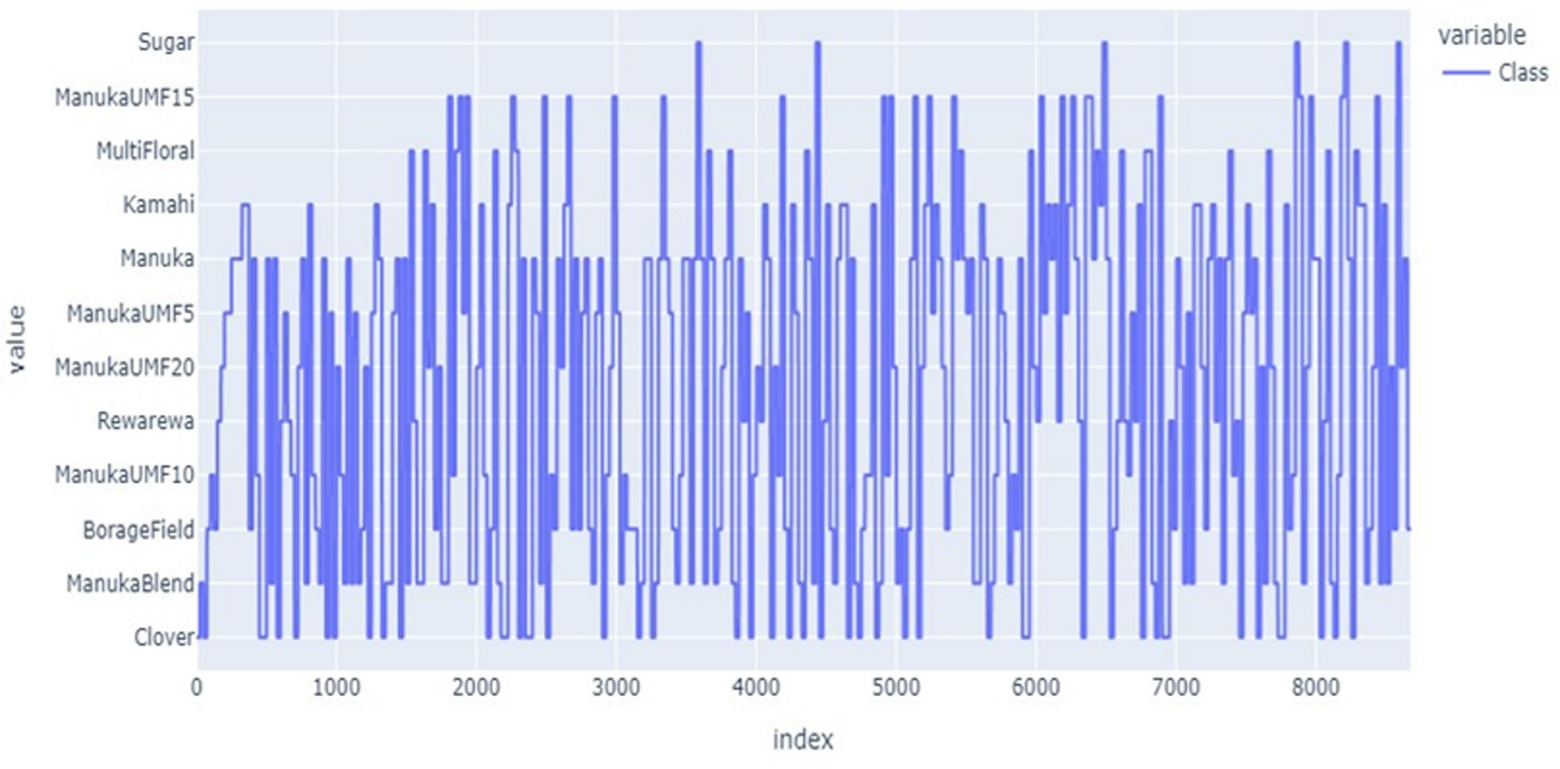
Class features after hyperparameter tuning applied.

As Fig 11 indicates, we apply hyper-parameter tuning on the features that are fed into the algorithm to enhance the loop of model learning to find the set of hyper-parameters leading to the lowest error on the validation set. The index refers to the row indices of the data frame used in the study. These indices are typically sequential integers that identify each row of the data frame.

**Fig 12:**
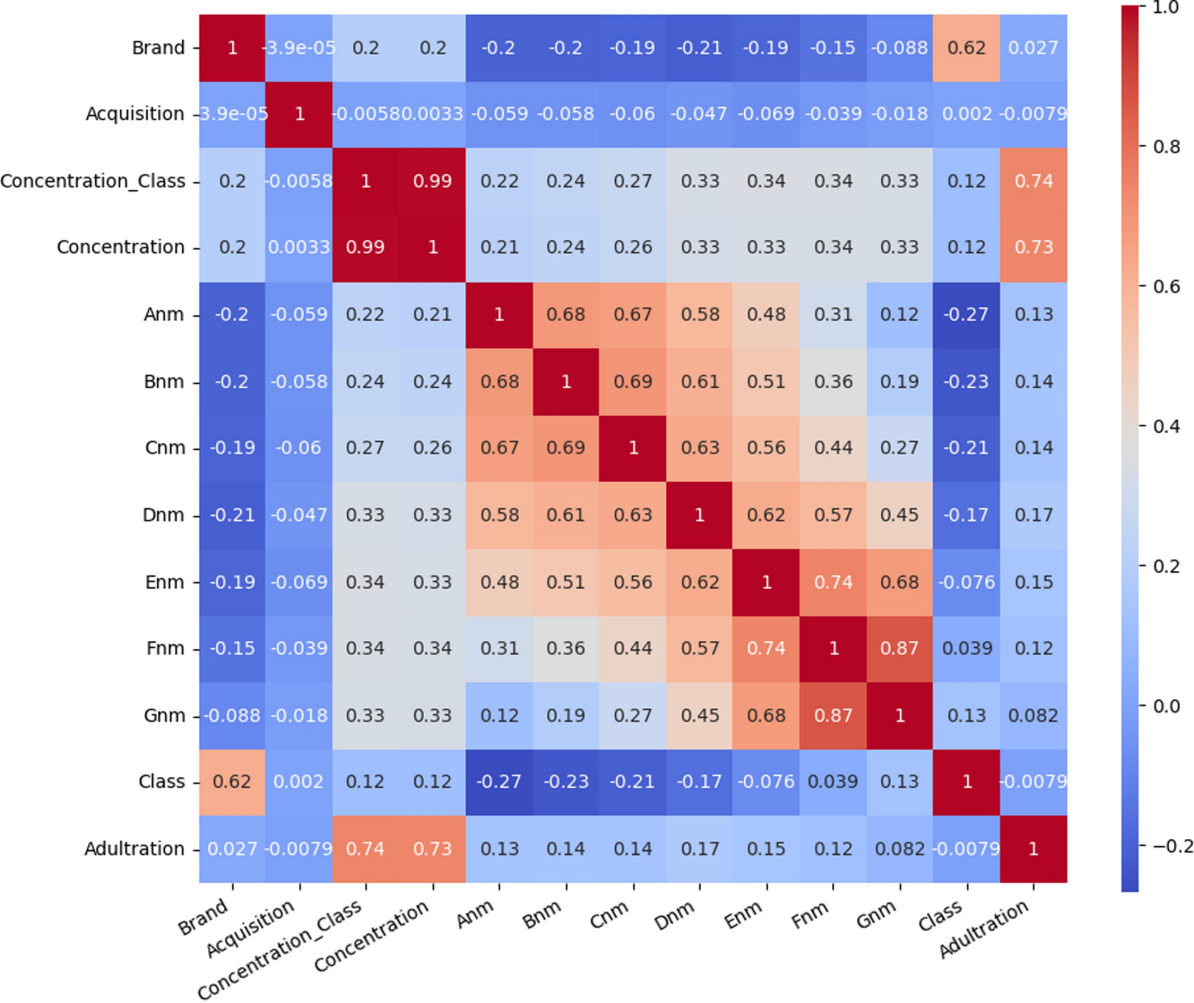
Confusion matrix of cleaned data.

The resulting confusion matrix is presented in Fig 12, the x-axis labels Anm, Bnm, Cnm, Dnm, Enm, Fnm, and Gnm, represent the wavelength bands captured by the hyperspectral imaging system, expressed in nanometers (nm). These labels denote the specific wavelengths at which spectral data were collected. The analysis conducted using these labels aims to determine how the error rate of the classification model fluctuates with different wavelength bands. By inspecting the error rate across various spectral features, we can pinpoint which wavelengths are most informative for detecting adulteration in honey. Essentially, these labels facilitate the understanding of the relationship between the spectral characteristics of honey samples and the accuracy of the classification model. Identifying crucial spectral features associated with adulteration enables us to enhance the effectiveness of our detection methods and develop more robust quality assurance techniques for honey products.

## Discussion

A study shows that machine learning and hyperspectral imaging can accurately identify if honey has been adulterated, with over 98% classification accuracy. The study found that between 5% and 10% of adulterated honey samples are misclassified, with C1 Clover honey being the most frequently misclassified. Manuka honey performed well in the multi-class problem due to its rich flavor and color content. Mislabeled classes include pure honey without tampering, with 25% adulterated honey mistaken for 10% and 50% adulterated honey.

The results and comparisons of different classifiers after data training and testing are presented. The findings in Table 3 show that the artificial neural network (ANN) is a stronger method for detecting honey adulteration, with a high accuracy of 98.8%.

**Table 3:** Accuracy evaluation report.

Model	Precision	Recall	f1-score	Accuracy
**ANN**	0.955	0.965	0.959	0.979
**SVM**	0.914	0.946	0.929	0.974
**KNN**	0.921	0.924	0.922	0.971
**RFR**	0.906	0.945	0.925	0.973
**DT**	0.940	0.953	0.946	0.976

**Fig 13:**
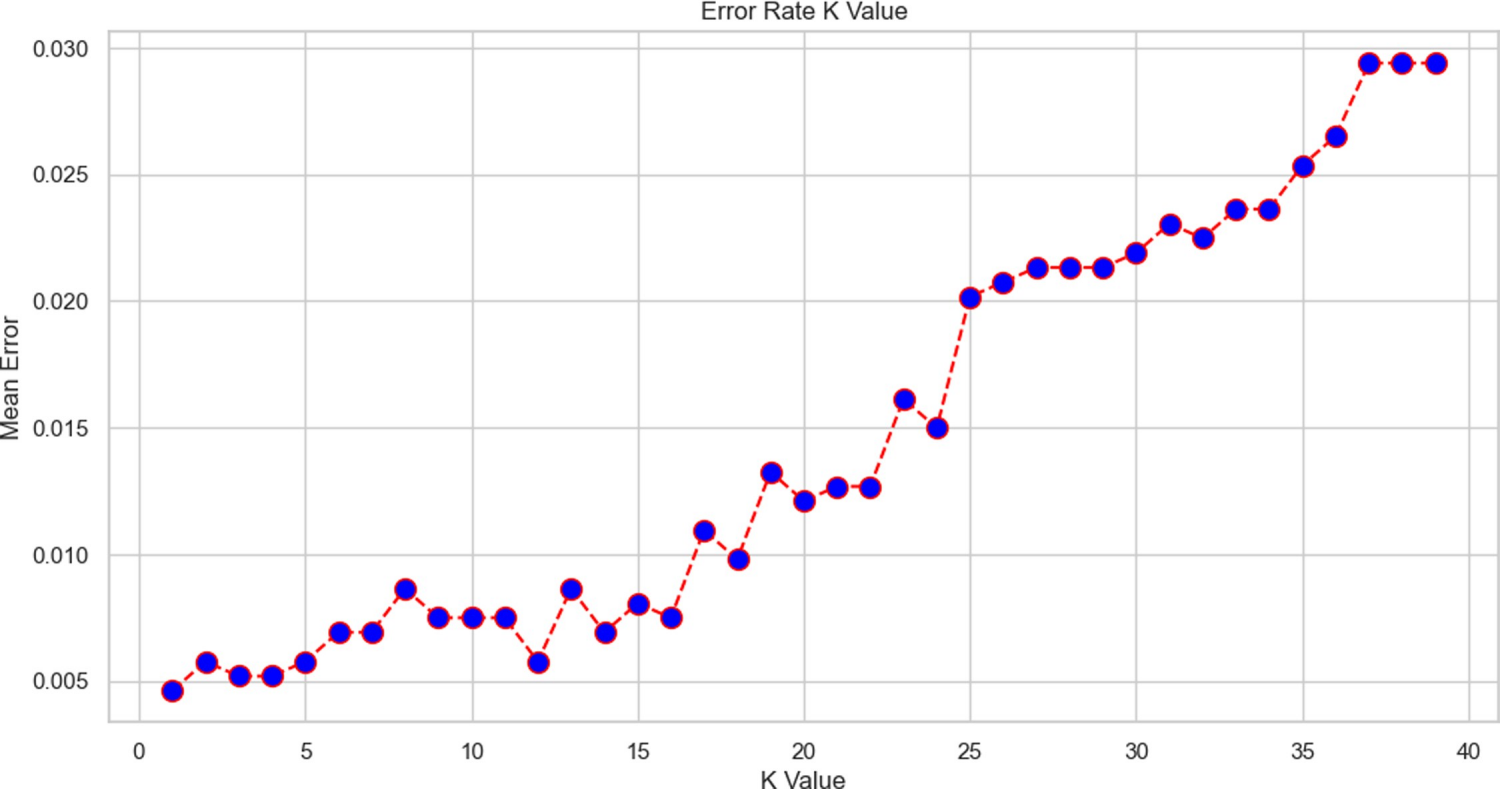
Error Rate with K Value.

We have compared the accuracy of four distinct machine learning models in the aforementioned Table 4. It’s evident that the ANN has the highest accuracy of all the models, but training takes a while. While random forest regressor and KNN are the lowest accuracy percentage models, SVM, and decision tree are roughly the same lower in terms of accuracy percentage. Fig 13 shows how the accuracy of KNN declines as K values increase.

**Table 4:** Accuracy evaluation report after enhancement using hyperparameter tuning.

Model	Precision	Recall	f1-score	Accuracy
**ANN**	0.985	0.995	0.989	0.989
**SVM**	0.934	0.956	0.944	0.945
**KNN**	0.931	0.924	0.927	0.928
**RFR**	0.936	0.935	0.935	0.937
**DT**	0.948	0.958	0.952	0.953

Table 4 shows the results of binary classification between non-adulterated honey and all concentrations of adulterated honey. Acc is the performance accuracy between zero and one. Std is the standard deviation of the cross-validation results. F1 refers to the average F1 score for all classes. The results show that even classical techniques such as ANN can perform well. The best algorithm achieves above 98% accuracy on this problem, making them a valuable addition to fraud protection measures already in place for honey adulteration.

A quick and reliable method of detecting adulterated honey, even at low percentages, is beneficial for exporting and importing honey products with reliable quality assurance.

The results from Table 4 show that there is some improvement from hyperparameter tuning and data pruning.

This research aims to use machine learning to detect adulteration in honey. Most classifiers benefit from the hyperparameter technique, with the hyperparameter method outperforming the others in accuracy and F1 scores. However, tuning the hyperparameters does not affect the decision tree classifier’s performance. The findings show that hyperspectral imaging and machine learning can accurately detect sugar syrup in honey. This system would be an excellent addition to the existing honey quality assurance methods. The performance evaluation is restricted to honey in the dataset, but as the dataset grows, a more general fraud detection system can be developed.

Misclassifications in the binary classifier are mostly caused by 5% and 10% adulterated honey samples, which affect C1 Clover honey. The study employs the largest data set with the most diverse honey types, yielding an accuracy of 97.6% for binary adulteration detection and 98.9% for multi-class adulteration classifications. A previous study reported a higher accuracy of 99.2%, but this result is not statistically valid due to the use of only 16 samples for four different concentrations and four honey types.

## Conclusions

In conclusion, our study highlights the importance of food safety, particularly in addressing the issue of honey adulteration, which poses significant risks to public health and undermines consumer trust. Leveraging machine learning (ML) technologies, including artificial neural networks (ANN), support vector machines (SVM), K-nearest neighbors, random forests, and decision trees, we conducted a comparative analysis to classify honey samples based on hyperspectral imaging data. Our findings underscore the potential of ML and hyperspectral imaging in accurately detecting adulterated honey, achieving classification accuracies exceeding 98%. Notably, the ANN model demonstrated superior performance, further enhanced through hyperparameter tuning. While promising, our study acknowledges limitations, including dataset diversity constraints and the need for further research to validate our findings in real-world settings. Despite these challenges, our research contributes to the ongoing efforts in food safety by providing valuable insights into combating honey adulteration. The integration of ML and hyperspectral imaging offers a promising avenue for developing robust quality assurance measures in the honey industry, thereby safeguarding consumer health and trust.

Looking ahead, future research directions may include expanding datasets to encompass a broader range of honey samples, refining modeling techniques, and exploring complementary analytical methods to enhance detection capabilities. Through continued innovation and collaboration, we can advance the development of reliable tools for ensuring the integrity and authenticity of honey products, thereby upholding food safety standards and consumer confidence.

## References

[1] King T, Cole M, Farber JM, Eisenbrand G, Zipper C, Sperber W, et al. Food safety for food security: Relationship between global megatrends and developments in food safety. Trends Food Sci Technol. 2017;68:160–175.

[2] Moore JC, Spink J, Lipp M. Development and application of a database of food ingredient fraud and economically motivated adulteration from 1980 to 2010. J Food Sci. 2012;77(4):R118–R126. doi: 10.1111/j.1750-3841.2012.02657.x 22486545

[3] Choudhary A, Gupta N, Hameed F, Choton S. An overview of food adulteration: Concept, sources, impact, challenges and detection. Int J Chem Stud. 2020;8(1):2564–2573.

[4] Maione C, Barbosa F Jr, Barbosa RM. Predicting the botanical and geographical origin of honey with multivariate data analysis and machine learning techniques: A review. Comput Electron Agric. 2019;157:436–446.

[5] Fakhlaei R, Selamat J, Khatib A, Zaini HM, Babadi AA, Manap MY, et al. The toxic impact of honey adulteration: A review. Foods. 2020;9(11):1538. doi: 10.3390/foods9111538 33114468 PMC7692231

[6] Zábrodská B, Vorlová L. Adulteration of honey and available methods for detection ‐ A review. Acta Vet Brno. 2015;83(10):85–102.

[7] Hu S, Ren D, Wei Q, Chen X, Wu J, Lu M, et al. Raman spectroscopy combined with machine learning algorithms to detect adulterated Suichang native honey. Sci Rep. 2022;12(1):3456. doi: 10.1038/s41598-022-07222-3 35236873 PMC8891316

[8] Ahmed E, Letta A, Noor S. Book Recommendation Using Collaborative Filtering Algorithm. Appl Comput Intell Soft Comput. 2023;2023.

[9] Goyal K, Kumar P, Verma K. Food adulteration detection using artificial intelligence: A systematic review. Arch Comput Methods Eng. 2022;29(1):397–426.

[10] Oroian M, Olariu V, Ropciuc S. Influence of adulteration agents on physico-chemical and spectral profile of different honey types. Int J Food Eng. 2018;4:66–70.

[11] Guler A, Aktumsek A, Varol GA, Onder H, Biyik S, Kocaokutgen H, et al. Comparing biochemical properties of pure and adulterated honeys produced by feeding honeybees (Apis mellifera L.) colonies with different levels of industrial commercial sugars. Kafkas Üniversitesi Vet Fakültesi Derg. 2017;23(2).

[12] Kelly JD, Petisco C, Downey G. Application of Fourier transform mid-infrared spectroscopy to the discrimination between Irish artisanal honey and such honey adulterated with various sugar syrups. J Agric Food Chem. 2006;54(17):6166–6171. doi: 10.1021/jf0613785 16910703

[13] Gallardo-Velázquez T, Osorio-Revilla G, Zuñiga-de Loa M, Rivera-Espinoza Y. Application of FTIR-HATR spectroscopy and multivariate analysis to the quantification of adulterants in Mexican honeys. Food Res Int. 2009;42(3):313–318.

[14] Ruiz-Matute AI, Rodríguez-Sánchez S, Sanz ML, Martínez-Castro I. Detection of adulterations of honey with high fructose syrups from inulin by GC analysis. J Food Compos Anal. 2010;23(3):273–276.

[15] Kumaravelu C, Gopal A. A review on the applications of Near-Infrared spectrometer and Chemometrics for the agro-food processing industries. 2015 IEEE Technol Innov ICT Agric Rural Dev. 2015:8–12.

[16] Siddiqui AJ, Musharraf SG, Choudhary MI, Rahman A, Ullah R, Ahmed A, et al. Application of analytical methods in authentication and adulteration of honey. Food Chem. 2017;217:687–698. doi: 10.1016/j.foodchem.2016.09.001 27664687

[17] Wu X, Tang J, Huang W, Li Y, Chen L, Han B, et al. Botanical origin identification and adulteration quantification of honey based on Raman spectroscopy combined with convolutional neural network. Vib Spectrosc. 2022;123:103439.

[18] Cuevas-Glory LF, Pino JA, Santiago LS, Sauri-Duch E. A review of volatile analytical methods for determining the botanical origin of honey. Food Chem. 2007;103(3):1032–1043.

[19] Trifković J, Andrić F, Ristivojević P, Guzelmeric E, Yesilada E. Analytical methods in tracing honey authenticity. J AOAC Int. 2017;100(4):827–839. doi: 10.5740/jaoacint.17-0142 28527183

[20] Nagamalla V, Saikiran K, Kethireddy SS, Reddy VR. Detection of adulteration in food using recurrent neural network with internet of things. J Food Qual. 2022;2022:1–11.

[21] Han J, Li T, He Y, Gao Q. Using Machine Learning Approaches for Food Quality Detection. Math Probl Eng. 2022;2022.

[22] Dave VS, Dutta K. Neural network based models for software effort estimation: a review. Artif Intell Rev. 2014;42(2):295–307.

[23] Abiodun OI, Jantan A, Omolara AE, Dada KV, Mohamed NA, Arshad H. State-of-the-art in artificial neural network applications: A survey. Heliyon. 2018;4(11):e00938. doi: 10.1016/j.heliyon.2018.e00938 30519653 PMC6260436

[24] Mozaffari A, Emami M, Fathi A. A comprehensive investigation into the performance, robustness, scalability and convergence of chaos-enhanced evolutionary algorithms with boundary constraints. Artif Intell Rev. 2019;52:2319–2380.

[25] Alzubi OI, Alzubi JA, Alweshah M, Qiqieh I, Al-Shami S, Ramachandran M. An optimal pruning algorithm of classifier ensembles: dynamic programming approach. Neural Comput Appl. 2020;32:16091–16107.

[26] Dutta S, Bandyopadhyay SK. Employee attrition prediction using neural network cross validation method. Int J Commer Manag Res. 2020;6(3):80–85.

[27] Khera SN, Divya. Predictive modelling of employee turnover in Indian IT industry using machine learning techniques. Vision. 2018;23(1):12–21.

[28] Kramer O, Kramer O. K-nearest neighbors. Dimens Reduct with unsupervised nearest neighbors. 2013:13–23.

[29] Breiman L. Random forests. Mach Learn. 2001;45:5–32.

[30] Biau G, Scornet E. A random forest guided tour. Test. 2016;25:197–227.

[31] Tan PN, Steinbach M, Kumar V. Introduction to data mining. Pearson Education India; 2016.

[32] Markey J. Using Decision Tree Analysis for Intrusion Detection: A How-To Guide. Sans Institute. 2011.

[33] Maimon OZ, Rokach L. Data mining with decision trees: theory and applications. World scientific; 2014.

[34] Çiğışar B, Ünal D. Comparison of data mining classification algorithms determining the default risk. Sci Program. 2019;2019:1–10.

[35] Powers DM. Evaluation: from precision, recall and F-measure to ROC, informedness, markedness and correlation. arXiv Prepr arXiv. 2020;2010:16061.

[36] Ahmed E, Abdu K. Maize Disease Detection using Color Cooccurrence Features. 2023.

